# Synthetic Data in Quantitative Scanning Probe Microscopy

**DOI:** 10.3390/nano11071746

**Published:** 2021-07-02

**Authors:** David Nečas, Petr Klapetek

**Affiliations:** 1 Central European Institute of Technology, Brno University of Technology, Purkyňova 123, 61200 Brno, Czech Republic; pklapetek@cmi.cz; 2 Czech Metrology Institute, Okružní 31, 63800 Brno, Czech Republic

**Keywords:** nanometrology, data synthesis, scanning probe microscopy

## Abstract

Synthetic data are of increasing importance in nanometrology. They can be used for development of data processing methods, analysis of uncertainties and estimation of various measurement artefacts. In this paper we review methods used for their generation and the applications of synthetic data in scanning probe microscopy, focusing on their principles, performance, and applicability. We illustrate the benefits of using synthetic data on different tasks related to development of better scanning approaches and related to estimation of reliability of data processing methods. We demonstrate how the synthetic data can be used to analyse systematic errors that are common to scanning probe microscopy methods, either related to the measurement principle or to the typical data processing paths.

## 1. Introduction

Scanning probe microscopy (SPM) is one of the key techniques in nanometrology [1,2,3]. It records the sample topography—possibly together with other physical or chemical surface properties—using forces between the sharp probe and sample as the feedback source. SPM has an exceptional position among nanometrological measurement methods when it comes to topography characterisation. Apart of versatility and minimum sample preparation needs its main benefit in nanometrology is the simple metrological traceability compared to some other microscopic techniques. Achieving a very high spatial resolution is, however, a demanding task and instruments are prone to many different systematic errors and imaging artefacts. The goal of nanometrology is to provide metrological traceability, i.e., an unbroken chain of calibrations starting from top level etalons, down to the microscopes. An important part of this task is expressing the measurement uncertainty, which means understanding these systematic errors and artefacts and which is one of the crucial aspects of transition from qualitative to quantitative measurements.

Measurement uncertainty in microscopy consists of many sources and to evaluate them we usually need to combine both theoretical and experimental steps. This includes measurements of known reference samples, estimation of different influences related to the environment (thermal drift, mechanical, and electrical noise), but also estimation of impact of data processing as the raw topography (or other physical quantity) signal is rarely the desired quantity. One of the approaches to analyse the uncertainty is to model the imaging process and data evaluation steps on the basis of known, ideal, data. Such an approach can be used at different levels of uncertainty analysis —at whole device level this is related to the virtual SPM construction [4,5], trying to incorporate all instrumentation errors into a large Monte Carlo (MC) model for uncertainty propagation. There are, however, many finer levels where ideal, synthesised, data can be used and which are becoming more popular. As one of the software tools that can be used for both SPM data synthesis and analysis, the open source software Gwyddion [6,7], was developed by us and is being used already by different authors for artificial data synthesis tasks, we would like to review the state of artificial data use in SPM.

Artificial data can be used for a multitude of purposes in the SPM world. Starting with the measurement aspects, they can be used to advance scanning methodology, for example adaptive scanning, like in Gwyscan library [8] focusing on sampling data for optimal collection of statistical information about roughness. Similarly, generated data can be used for development of even more advanced sampling techniques, e.g., based on compressed sensing [9,10]. In contrast to measured data, generated datasets allow estimating the impact of different error sources on the algorithm performance in a more systematic manner. Similarly, generated data were used for analysis of uncertainties related to the instrumentation, like better understanding of effects related to the feedback loop [11] in tapping mode measurements.

Going even further in the instrumentation direction, generated data can be used to create novel and advanced samples or devices for metrology purposes. Generated data were used for driving a calibration platform that is mimicking the sample surface by moving up and down without lateral movement, either for creating a virtual step height or defined roughness sample [12,13]. Such approach is one way how to provide traceability to commercial microscopes that have no built-in interferometers or other high level metrology sensors. Synthetic surface model was also used to create real samples with know statistical properties, e.g., self-affine surfaces designed for cell interaction studies using two-photon polymerisation [14] or isotropic roughness designed for calibration purposes using by a focused ion beam [15,16].

The largest use of artificial data is, nonetheless, in the analysis of data processing methods, where they can serve for validation and debugging of the methods and to estimate their sensitivity and reliability. They were used to test data processing methods and to determine uncertainties related to the imaging process, namely tip convolution and its impact on different quantities. The impact of tip convolution on statistic properties of columnar thin films [17,18], fractal properties of self-affine surfaces [19,20], and size distribution of nanoparticles [21] were studied using entirely synthetic data. Novel approaches for tip estimation using neural networks were developed using artificial rough data [22] and methods for using neural networks for surface reconstruction to remove such tip convolution errors were developed using simulated patterned surfaces [23]. Algorithms for double tip identification and correction in AFM data were tested using synthetic data with deposited particles of different coverage factors [24]. Simple patterns combined with Gaussian roughness were used for development of non-local means denoising for more accurate dimensional measurements [25]. Combined with real measurements, synthetic data were used to establish methodology for spectral properties evaluation on rough surfaces [26] and spectral properties determination from irregular regions [27]. Synthetic data were used to help with results interpretation and for finding relationship between mound growth and roughening of sputtered films [28]. Combination of real and synthetic datasets was used for determination of impact of levelling on roughness measurements [29] and for development of methods for splitting instrument background and real data in analysis of mono atomic silicon steps [30]. They were used to develop methods for grating pitch determination [31] and reliability measures for SPM data [32]. In the area of data fusion, they were used for construction of methods for low and high density data fusion in roughness measurements [33]. Even more general work was related to the impact of human users in the SPM data processing chain on measurement uncertainty [34].

Artificial data can also be used to estimate the impact of topography on other SPM channels. Most of the techniques used for measuring other physical quantities than length are influenced by local topography, creating so called ‘topography artefacts’. To study them one can create a synthetic surface, run a numerical calculation of the probe–sample interaction and simulate what would be the impact of a particular topography on other data sources. This approach was used for simulation of C-AFM on organic photovoltaics on realistic morphologies similar to experiment [35], for simulation of topography artefacts in scanning thermal microscopy [36] and for simulation of impact on lateral forces in mechanical data acquisition [37].

In this work we review the methods used for generation of synthetic data suitable for different SPM related tasks and give examples how these methods can be run in Gwyddion open source software. Many of the presented methods are more general and can be applied also to the analysis of other experimental techniques based on interaction with surface topography, for example to study the impact of line edge roughness in scatterometry of periodic structures [38].

## 2. Artificial SPM Data

Artificial SPM data can be produced by many different procedures. Some of them attempt to capture details of the physical and chemical processes leading to the surface topography formation. Others are designed to mimic the final shape of the structures without any regard to the underlying processes. Frequently the algorithms lie between these two extrema, trying to preserve a physical basis while being fast enough for practical purposes. In the following we group the methods to several broad classes, more or less corresponding to the character of the generated data and types of phenomena simulated. We give particular attention to models implemented as Gwyddion modules—this is noted by giving the module name in italics.

Results of the data synthesis algorithms presented below are usually height fields—regular arrays of height data in which for each coordinates (x,y) there is only one *z* value. This is different from general 3D data, but it is also the standard SPM measurement output.

### 2.1. Geometrical Shapes and Patterns

Well-defined geometrical objects are frequent in nanotechnological samples, whether coming from microelectronics, MEMS or other fields. They need to be measured and reconstructed in SPM simulations. Both can be done within a single framework. Fitting shapes to measured topographical data is the inverse (i.e., harder) problem to their construction from given parameters. Therefore, construction comes more or less free with shape fitting. This is also the approach taken in Gwyddion, where the *fit shape* module can be also used directly to create artificial AFM data representing the ideal topographies.

Modelling of ideal shapes like steps, trenches, pyramids or cylinders usually only involves elementary geometry and the model is just z=f(x,y), where *f* is an explicit function such as $z={\left(x^{2}+y^{2}\right)}^{1/2}$ for a cone (cones with other parameters are then created by coordinate transformations, such as rotation, scaling, or folding). Overlapping neighbour features and faceted shapes can be modelled using $f=\min_{i}f_{i}$ or $f=\max_{i}f_{i}$, where $f_{i}$ are simpler functions (i=1,2,…). Still, some geometrical models can become rather involved, for example for rounded indentation tips [39].

The microscope tip is an extremely important geometrical shape in SPM. Widely used models include cylinder with cone and spherical cap [40], pyramid with spherical cap [41], hyperboloid [42,43], and paraboloid [44]. Parametrisable tip models are needed namely when mechanical properties are evaluated from force-distance curve measurements; models such as linearly interpolated tip or *n*-quadratic sigmoidal are then used [45]. Different tip models can be created using Gwyddion *model tip* module. More irregular tips can be produced by using any other synthesis module, e.g., particle deposition and cutting a suitable part of generated surface. Although true geometrical shapes are used in detailed simulations [46], commonly the tip shape is discretised, i.e., approximated as a height field, in particular when subsequent operations are defined in the pixel representation [47,48,49].

Furthermore, most SPM calibration samples are regular geometrical patterns. Some of these patterns may not be common in applications—however, they are obviously important in nanometrology itself. These patterns include various types of one-dimensional gratings (present in HS-20MG, HS-100MG, HS-500MG, TG series, 301BE, 292UTC, MRS-3, MRS-4, MRS-6, TGZ1, TGZ2, TGZ3, TGG1, TGF11, TGX series, 70-1D, 150-1D, 300-1D, 700-1D, 145TC, SGN01, SHS, ANT, MetroChip), two-dimensional arrays of rectangular or circular holes (HS-20MG, HS-100MG, HS-500MG, TGX series, MRS-3, MRS-4, MRS-6, SHS, ANT, MetroChip), two-dimensional pillar arrays (HS-20MG, HS-100MG, HS-500MG, TGQ1, flexCG120, TGX series, 300-2D, 150-2D, 700-2D, MetroChip, SNG01), concentric squares, L-shapes or rings (ANT, MRS-3, MRS-4, MRS-6, SHS, MetroChip), staircases (Sic/0.75, SiC/1.5, STEPP), chessboard (TGX1, MetroChip, UMG01, UMG02), Morse code/compact disc-like (750-HD), or the Siemens star pattern (ANT). Recently the silicon lattice step was adopted as a secondary realisation of the metre, in particular for nanometrology [50,51]. The corresponding calibration structures have the form of amphitheatres formed by single atom steps [30,51].

**Figure 1:**
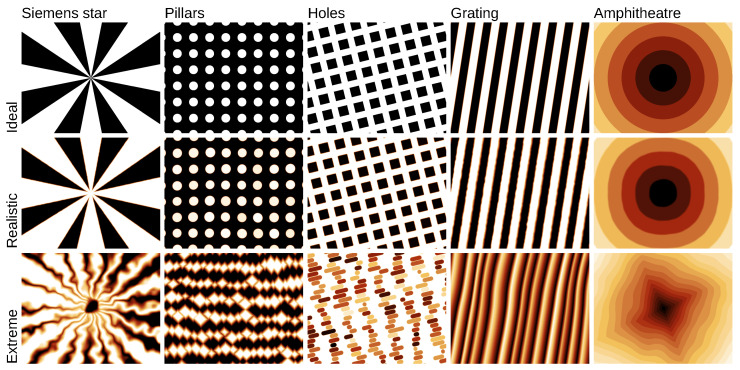
Selected examples of regular geometrical shapes generated by the *pattern* module. The **top row** (Ideal) depicts the ideal models. The **middle row** (Realistic) shows slightly non-ideal shapes exhibiting variability, line roughness or deformation. The **last row** (Extreme) illustrates the expressive power of the models using extreme variability and odd settings.

The patterns often have certain dimensions which are precise and intended for calibration, whereas other dimensions are not guaranteed. For instance usually the period of a grating (or its inverse, the pitch) is specified. Its other parameters, such as fill ratio, height, or side slopes are unspecified. Artificial data generation methods need to reflect this. Gwyddion’s *pattern* module creates regular patterns with exactly specified periods. Other parameters, such as width, height, slope, or placement of features can be also perfectly regular—or varied, but the variation is local, not disturbing the pitch. A subset of patterns corresponding to common calibration samples is illustrated in Figure 1. Realistic shapes require adding defects such as surface and line roughness. These will be discussed in Section 2.4.

**Figure 2:**
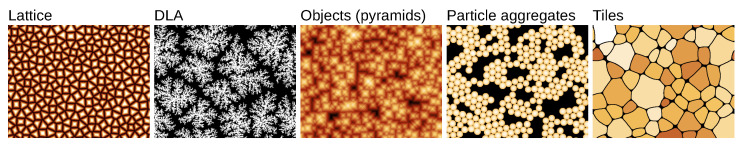
A slightly disordered snub square lattice (*lattice*), diffusion-limited aggregation (*diffusion*); random pyramidal surface (*objects*); settling of particles on surface (*particles*); and random smooth tiles (*tiles*).

Finally, it is useful to generate regular lattices, for instance to represent atomic lattices. Of course, a lattice alone is not sufficient to simulate a technique such as STM. Solid state physical computations are necessary (generally DFT-based) [52,53,54] together with Tersoff–Hamann approximation for the STM signal [55]. However, the investigation of a data processing method behaviour may not require ab initio results as input data and lattices can be useful in other contexts. Artificial data can be then produced by first generating points corresponding to a regular and semi-regular tilings, Penrose tilings [56], the Si 7×7 surface reconstruction or any other interesting two-dimensional pattern. The point locations can be randomly or systematically disturbed. The actual neighbourhood relations are then obtained using Delaunay triangulation [57,58] and Voronoi tessellation [58,59] and quantities, such as distance from the nearest point or nearest boundary are used to render the image (*lattice*, Figure 2).

### 2.2. Deposition and Roughening

Random roughness and nearly-stochastic surface textures are ubiquitous in material science as they can arise from almost any processing—for instance deposition, etching, mechanical contact, or crystallisation. The influence of roughness increases at nanoscale where its characteristic dimensions can be comparable to the dimensions of the objects and it can even become the dominant effect influencing surface properties [60,61,62,63,64]. Frequently it is crucial to include it in simulations.

The importance of surface roughness is reflected by the extensive literature published on this topic, including many approaches and algorithms for simulation of roughening processes [65,66]. Models of growth of rough surfaces during depositions are among the most studied. At nanoscale, deposition is probably the most common roughening process (whereas at macroscale subtractive processes such as machining are more common). Roughness growth models are also of significant theoretical interest as the scaling exponents are related to the underlying physical processes [65].

Most practical growth models are discrete, i.e., realised in a grid, usually one matching image pixels, and formulated in terms of individual pixel values —some can even be considered cellular automata. The height dimension can be treated differently with respect to discretisation, value scale, etc. The distinction between 3D and 2+1-dimensional models is not always clear in this case. Still, they generate topographical images, i.e., height fields. The second major difference from the previous section is that most models considered in this and the following sections are inherently random. This can mean growth and roughening simulation using stochastic partial differential equations (PDE), such as the Kardar–Parisi–Zhang (KPZ) equation [65,67,68,69] $\partial_{t}z=\nu \Delta z+\left(\lambda /2\right){\left(\nabla z\right)}^{2}+\eta$. Parameter ν and λ characterise the surface tension and lateral growth; η is uncorrelated white Gaussian noise. A KPZ modification known as the Kessler–Levine–Tu (KLT) model [70] has been used for simulation of the etching process producing rough light-trapping surfaces [71]. However, even more commonly the model is an MC simulation of some kind of process, at least in a loose sense. Both approaches produce random instances of surfaces by sampling a probability space.

**Figure 3:**
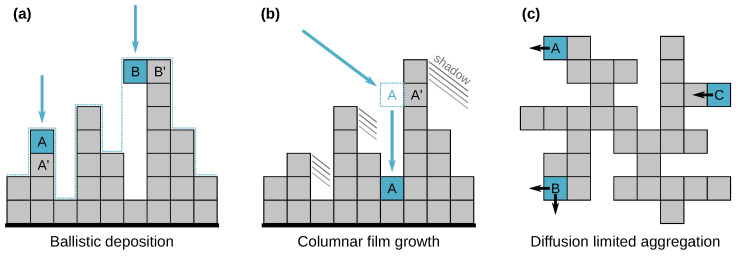
Deposition models: (**a**) In ballistic deposition the falling particles A and B stick to the highest points of contact (A’ and B’), possibly creating voids (however, the only the top surface, shown as dotted line, defines the simulation state). (**b**) Oblique incidence in columnar film growth creates as shadowing effect as the incoming particle encounters the tallest column (at A’); it then relaxes to an energetically preferable site; (**c**) In the top view of diffusion limited aggregation simulation, particle A has to break one bond to move in indicated direction while B has to break two—particle C has to pass the Schwoebel barrier to move to second layer.

The simplest MC deposition model is random deposition [65,72], in which small particles fall independently onto the surface at random positions, increasing the height at that position. It produces uncorrelated noise with Gaussian distribution, which can be easily generated directly (see also noise models in Section 2.4). When the particles are allowed to relax to the lowest neighbour position, lateral correlations appear—nevertheless with scaling exponents α=β=0 in 2D (the scaling is logarithmic). The simplest classical model with an interesting behaviour is thus ballistic deposition (*Ballistic*) [65,73,74], in which the particles immediately stick to the surface at the first contact—see Figure 3a for an illustration. The particle positions are generated randomly with a uniform distribution over the area. Although ballistic deposition is simple, it produces self-similar surfaces in the same universality class as the KPZ equation and has been used for modelling of colloidal aggregates [75]. It can also be seen as the base for other models. Models considering additional particle behaviour after it touches the surface have successfully reproduced a number of real phenomena. A variety of models have been proposed for molecular beam epitaxy, with different relaxation rules, taking into account diffusion and possibly desorption [65,76,77]. The growth of columnar films can be reproduced if the incident particles do not fall vertically, but at random oblique angles, creating a shadowing effect (*columnar*) [65,74,78]. After hitting the surface the particle relaxes locally to a lower, energetically preferably position (Figure 3b, see also Figure 7 for an example).

On the other hand, if particles can travel long distances across the surface to find an energetically preferable site, this correspond to the DDA type of models (deposition, diffusion, and aggregation), which reproduce structures seen in sub-monolayer deposition, as well as other structures formed by particle aggregation [65,67,74]. In the diffusion-limited aggregation (DLA) model simulated particles can hop between surface sites, facing a barrier $E_{0}$. Neighbour particles increase the barrier by additional energy $E_{N}$, making dimers and larger clusters unlikely to break (Figure 3c). Using a Metropolis–Hastings type algorithm [79,80], the effect of energy barriers is the reduction in hopping probabilities by factor $\exp(-\Delta E/k_{B}T)$ for a barrier ΔE>0; $k_{B}$ and *T* being the Boltzmann constant and temperature. The atomistic simulation can also include a non-zero probability of passing the Schwoebel barrier, allowing particles to move between layers [81,82] (*diffusion*, Figure 2). Even models mentioned in the previous paragraph often exhibit different sub-monolayer and multilayer growth regimes, with a transition between them. The spectrum of phenomena observed in sub-monolayer and few-layer film growth is surprisingly rich and a variety models have been used to study specific processes, such as island ripening processes and roughening transitions. [65,83,84,85,86].

The deposition models have inspired several other random surface texture generation methods. A texture formed by random protrusions of given shape is generated by the following method (*objects*, Figure 2). A shape with finite support, for instance a pyramid, is generated at a random location. Surface minimum *m* over its support Ω is found: $m=\min_{i\in \Omega }\left\{z_{i}\right\}$, where *i* indexes the pixels with current heights $z_{i}$. The surface is then modified $z_{i}\to \max\left(z_{i},m+h_{i}\right)$, where $h_{i}$ is the pyramid height at pixel *i*. The procedure is repeated until given coverage by the shapes is reached. This model has been used for instance for the modelling of pyramidal solar cell surfaces and can be used to reproduce the textures of TG1 or PA series tip sharpness calibration samples. For single-pixel features the model is equivalent to random deposition, but larger features give raise to lateral correlations. Numerical simulations in 2+1 dimensions suggest scaling exponents α=1/2 and z=2 which differ from both random deposition with relaxation and KPZ. Nevertheless, in practice the model is used in the sub-monolayer regime up to a few layers.

Several other models follow the same scheme of choosing a random object and location. The surface is modified by the placed object according to a local rule—usually the height increases, but holes can be created instead for ‘etching’. A slightly more sophisticated version, which places real 3D shapes instead of 2D functions, for instance ellipsoids or rods, but still simply sticking to the place they touch, has been implemented in Gwyddion (*pile up*, Figure 7).

An actual physical simulation of interacting 3D objects is used to reproduce self-organised conglomerates formed by the settling of larger particles on the surface [21]. In this case the 3D objects are relaxed using an integration of Newton equations similar to a molecular dynamics simulation [87]. Interactions between particles and between a particle and substrate are modelled using the Lennard–Jones potential. An Anderson thermostat is used to simulate the Brownian motion of the particles; in addition the nanoparticle velocities are damped during the computation to simulate the decreasing mobility. The Verlet algorithm is used to integrate the Newton equations. By stopping the algorithm before convergence it is possible to simulate a partial relaxation, often observed in practice. This model is used in *particles* and *rods* modules in Gwyddion (Figure 2 and also Figure 7). In the case of rods relaxation, each rod is represented as a rigid configuration of three spheres, using the Settle algorithm [88], which is sufficient for simulation of behaviour of rods of small aspect ratio.

### 2.3. Order and Disorder

**Figure 4:**
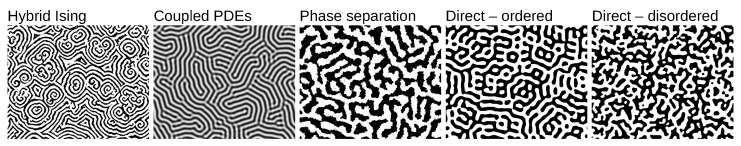
Self-organisation models: spiral waves, generated by hybrid Ising model (*domains*); Papillary lines type Turing pattern, produced by coupled PDEs (*coupled PDEs*); quenched disorder in phase separation obtained by simulated annealing (*annealing*); two patterns created by direct spectral synthesis and morphological post-processing (*phases*), with low and high disorder.

A different family of models is used to represent contrast patterns in non-equilibrium systems with spontaneous symmetry breaking and long-range organisation. Typical examples include waves in excitable media [89] which produce characteristic patterns found in diverse systems such as chemical reactions [90,91], vegetation patterns [92], propagating flame fronts [93] or cardiac tissue [94] (Figure 4); or static Turing patterns that play role in developmental biology [95,96,97] (Figure 4). More directly relevant for nanometrology are the patterns of magnetic domains in multilayers used as reference samples in magnetic force microscopy [98,99,100,101] or the morphology of phase separation [35]. We consider all models related to self-organisation, order–disorder transitions, phase separation and related phenomena to be part of this family.

A distinct feature of this class is that the data fields are non-topographical. Whether the values represent chemical concentrations or spin orientations, the computations occur in the xy plane with no notion of the third dimension.

An important classical model is quenched disorder in a regular solid solution, known also under many other names, such as Ising or lattice gas model [102,103,104,105,106,107]. It can reproduce patterns forming due to separation of phases or domains (a similar approach is also used to simulate the morphology of chains in a polymer-blend films [108]). Cooling from a high temperature, in which the system is in a disordered state, long-range correlations starts to appear as it nears the critical temperature $T_{c}$. A phase transition to ordered domains would occur at $T_{c}$ (in dimensions D>1, whereas for D=1 a gradual change is typical [105]). However, if the cooling is fast, the energy barriers can become large compared to $k_{B}T$ before the transition finishes and the system is frozen in an intermediate state. The patterns can be generated using simulated annealing, a Metropolis–Hastings type algorithm [79,80,109] (*annealing*, Figure 4). Each image pixel is in one of two (or possibly more) states. Random transitions, such as swapping two neighbour pixel states, can occur with probability $\min\left(1,\exp(-\Delta E/k_{B}T)\right)$ if the configuration energy increases by ΔE. The slow convergence for low *T* motivated the search of alternative algorithms (some of which are mentioned below).

For the formation of the eponymous patterns, Turing originally proposed a reaction–diffusion model [95]. However, since systems with local activation and long-range inhibition (LALI) are in general capable of forming such patterns [97,110,111], many other models have been presented over the years which exhibit similar behaviours. The standard modelling approach is coupled PDEs that for the reaction–diffusion model can be written $\partial_{t}c=D\;\nabla c+f\left(c\right)$, where c is the vector of component concentrations, *D* is a diagonal matrix of diffusion coefficients and f is a non-linear function describing the reaction kinetics (*coupled PDEs*, Figure 4). Two components are sufficient to reproduce a wide variety of interesting phenomena, although some types of behaviour require three or more components [112]. Alternative methods for patterns production have been proposed, often aiming to improve the efficiency of the long-range inhibition simulation. A so-called kernel based Turing model replaces the PDE with an explicit shape of activation–inhibition kernel convolved with the concentration variable [97].

Hybrid LALI models employ combinations of differential equations and cellular automata or other local discrete rules [113,114,115]. A hybrid non-equilibrium Ising model can be constructed by combining a discrete short-scale Ising model for two-state variable *u* with continuous slow inhibitor *v* described by a differential equation [113]. Variable *u* is updated using the standard scheme, with flipping probability $\min\left(1,\exp(-\Delta E/k_{B}T)/2\right)$. The state energies *E* are computed from the number of different neighbours *n* but also biased using *u* as E=Buv+Jn, where *B* determines the bias and *J* the interaction strength. Depending on the effective inhibitor diffusivity, *v* can be described either by a linear reaction-diffusion partial differential equation with macrogrid averaging (fast diffusion), or a local ordinary differential equation (non-diffusing inhibitor). In the second type (*domains*, Figure 4), *v* follows $\tau \dot{v}=-v-\nu +\mu u$, where ν and μ are bias and inhibitor strength, and τ is the characteristic time. This defines the relative timescales of *u* and *v* as they are alternately updated. The model has several regimes and can reproduce both the spiral waves and phase separation-like patterns.

Extensive simulations require generating large amounts of artificial data and models involving any kind of time evolution may be too time-consuming. Depending on the application, fast models which abandon the simulation path and just directly reproduce the basic features of the patterns may be preferable. An example is the model mimicking Turing pattern type textures of MFM calibration samples [100]. It is based on the peculiar frequency spectra in which one spatial frequency strongly dominates due to Turing instability [95]. The construction has two steps. Synthesis in the frequency domain provides data with the narrow frequency spectrum. Morphological post-processing then refines the local morphology to resemble more closely the real patterns (*phases*, Figure 4).

### 2.4. Instrument Influence

A special class of data synthesis methods models the various artefacts related to the measurement principle and measurement process. In the SPM world the most prominent type of modification is so called tip convolution, a distortion of measured morphology related to the fact that the SPM probe is not infinitely sharp. The resulting dataset is a convolution (mathematically, a dilation) of the probe and sample [47]. For a known probe the effect can be simulated using algorithms presented in Reference [47], producing simulated AFM result from true (ideal) topographical data.

Thermal drifts are present in nearly all the SPMs [116]. They are related to thermal expansion of different microscope components before the thermal equilibrium is reached after instrument start or when the temperature in the laboratory is not sufficiently stable. They can be simulated by adding some *x*, *y* and *z* components to the simulated data. Another source of distortion are scanning system imperfections. In open loop systems SPM scanners are subject to systematic errors related to the piezoelectric actuators hysteresis, non-linearity, and creep [117]. In closed loop systems errors arise from non-linearity of sensors and inaccuracy and linear guidance system imperfections [118].

**Figure 5:**
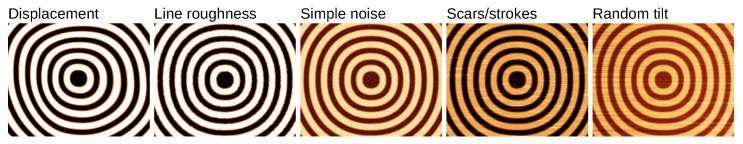
Simulated defects and scanning artefacts: slowly varying displacement field; quickly varying displacement field (simulating line roughness); simple pixel noise; scars/strokes; random tilt of scan lines. The undisturbed surface is always the same system of concentric super-ellipses.

A quite general technique for distortions in the xy plane is displacement field (*displacement Field*, Figure 5). Consider a vector field v(r) defined as a function of planar coordinates r=(x,y). The distorted image $z^{\prime }$ is created from original image *z* using $z^{\prime }\left(r\right)=z\left(r+v(r)\right)$ with suitable handling of z(r) for r falling outside the image (periodic, border value extrapolation or mirroring). A slowly varying v represents drift and similar systematic effects, for instance in xy plane by putting $v=R\left(-\phi \right)\left(r-r_{0}-b\right)-\left(r-r_{0}\right)$, where b and ϕ are shift and rotation with respect to centre $r_{0}$ (*R* denotes rotation matrix). The time dependencies of b and ϕ define the drift—in the simplest case of linear drift they can be taken proportional to c·r, where c is a constant vector formed by inverse scanning speeds. On the other hand, v varying on short scales can model non-instrumental effects, such as line and surface roughness [119]. Both are illustrated in Figure 5. There are many possible useful choices for v: explicit functions (polynomials), random Gaussian fields or other correlated noise, or even other images. We formulated the distortion for images and this is how it usually applied. Nevertheless, for explicit functions like those in Section 2.1 the displacement can be applied directly to the coordinates, without intermediate pixelisation.

Noise is ubiquitous and is related to different effects—noise in the electronic circuits, mechanical vibrations and feedback loop effects. The spectral properties of noise depend on its source, like 1/f noise being related to light fluctuation [120] or shot noise to detection of light beam reflected from cantilever that is frequency independent. Often it has some dominant frequencies, either related to the electrical sources from the power line, characteristic mechanical vibration frequencies of the tip-sample system, or acoustic noise from the environment [121]. In artificial data preparation, noise can be generated independently and added to the synthetic data in post-processing. For simple simulations independent noise in each pixel can be sufficient (*noise*, Figure 5). Correlated noise with given power spectrum is generated using spectral synthesis [74], i.e., constructing the Fourier coefficients with given magnitudes and random phases and using the inverse fast Fourier transform (FFT) to obtain the correlated noise (*spectral*, see Figure 9 for image examples). A special type of noise related to the feedback loop effects are scars/strokes, short segments of the scanned line that are not following the surface (*line noise*, Figure 5).

Another important type of noise related to the scanning process is the line noise, which causes shifts between individual lines scanned in the fast scanning axis that form the image. The source of this noise is not very well understood, but most probably it is a mixture of low frequency noise, drift, impact of changing the tip motion direction, and impact of tiny changes of the tip behaviour (contamination, tip wear, etc.). It can also be added to synthetic data using *line noise* in Gwyddion (see Figure 10b in Section 3.2 for an example).

An important error source in optical detection based SPMs (i.e., nearly all commercial systems) is the interference of light which misses cantilever and is reflected off the sample surface towards the beam deflection detector [122,123]. It can be modelled using simple geometrical optics [122]. However, in practice also diffraction effects can play a role as diffraction pattern can be often seen in the beam reflected from cantilever. The effect can be visible namely on very flat sample measurements, creating a pattern in the topography channel resembling interference fringes. This is usually a reason for re-aligning the laser position on the cantilever. However, residuals of this effect cannot be so easily noticed yet they still affect the topography measurements. In the simplest approximation this error source can be expressed as a harmonic function of the height and in Gwyddion can be added using *data arithmetic*. The procedure includes taking the surface, separating details and polynomial background out of it, calling the background b(x,y), adding Asin(4πb/λ) to it and merging it again with the details.

Feedback loop effects are related to undershoots and overshoots of the proportional-integral-derivative (PID) controller that is used to keep the probe–sample interaction constant via *z* motion of the tip or sample. These can be simulated for synthetic data by establishing a virtual feedback loop based on a model force-distance dependence and calculating the cantilever response using a suitable model, e.g., damped harmonic oscillator for tapping mode measurements [124] combined with modelling the time evolution of the feedback loop response. A simple feedback loop simulation is also included in Gwyddion (*PID*).

### 2.5. Further Methods

**Figure 6:**
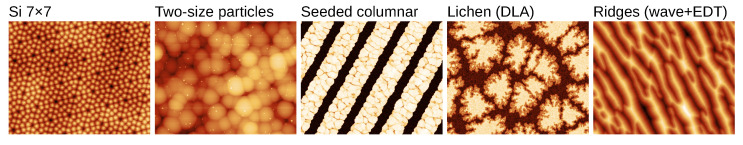
Multi-step constructions: Si 7×7 lattice with artefacts; ellipsoids of two different sizes; seeded columnar film growth; morphologically post-processed DLA; and sum of sine waves, also morphologically post-processed.

Preceding sections introduced several classes of surface and texture generation methods which are natural candidates for artificial SPM data because of physical or metrological reasons. However, the options are not limited to simply running them. Highly complex artificial data can be obtained by chaining several algorithms. In particular, ideal patterns are frequently combined with defect generators for realistic data. A generated precise geometrical pattern can be modified in sequence by added particles, displacement field, feedback loop effects, and line or point noise. Two such examples can be seen in Figure 6, a slightly distorted and uneven Si 7×7 surface reconstruction (with scanning artefacts added) and sequential ‘deposition’ of large and small particles (again with artefacts).

Gwyddion makes chaining particularly easy as all synthetic data generators can take an existing image as the starting point. An example is shown in Figure 6 where simulated columnar film growth was seeded by a grating generated by another module. This also allows using the same generator multiple times, for instance to create multi-scale patterns. Furthermore, the generators can be combined with other standard image and morphological processing methods—edge detection, opening, closing, or Euclidean distance transform (EDT) [125]. The ‘Lichen’ image in Figure 6 was created using DLA, post-processed with edge detection and correlated Gaussian noise. ‘Ridges’ originated as a simple sum of sine waves, which was then thresholded and EDT was applied to the result.

Combination of patterns generated at different scales is a standard technique in noise synthesis—although here usually only simple linear summation is used. Noise generators are an important classic category of which Section 2.4 listed a few but at least a few others need to be mentioned. The Perlin noise generator (and its newer alternative the simplex noise) [126,127] produce spline-based isotropic locally smooth noise. They are frequently used as multi-scale, combining outputs at different lateral scales to obtain a somewhat self-affine result. Stochastic midpoint displacement [74,127,128] (*Brownian*) is another a direct-space construction, in this case top-down. The generation starts with at a coarse scale with sparse grid. The grid is then progressively refined using midpoint interpolation with random value variation obeying a scaling law. The result approximates, to some degree, fractional Brownian motion. As an alternative to FFT-based spectral synthesis the Mandelbrot–Weierstrass method also sums a sine series, but with frequencies forming a geometric progression ($f_{n}∼c^{n}$) instead of an arithmetic one ($f_{n}∼n$) [74,129]. Other correlated noise generation approaches include sparse convolutions or wavelet synthesis [127].

SPM techniques are naturally connected to surfaces and processes on them. Modelling of processes occurring at material boundaries is a vast field. Many models have been developed for simulations at different scales that are typical in nanoscience—such as boundary front propagation in disordered media such as wetting, burning, or growth of bacterial colonies [65,130], dendrite formation simulated using the phase field method [131], ground surface topography by simulating material removal by active grains [132], pitting corrosion texture using stochastic cellular automata [133], or dune formation by random transportation of sand ‘slabs’ in a lattice [134]. Nevertheless, they may produce data useful for testing and evaluation of SPM data processing algorithms. This applies to pattern synthesis methods in general. Processes at different scales and with different underlying physical or chemical details give raise to similar structures—as was already noted in Section 2.2 and Section 2.3. When one is looking for a generator producing test data with particular spectral characteristics, connectivity, anisotropy or multi-scale properties, it can sometimes be found in unexpected places.

This extends even to procedural textures developed originally for computer graphics. Some do not have any basis in physics, such as maze generation using evolving cellular automata [135]—even though the results share some characteristics with Turing patterns. A round tile pattern can be created by recursively solving the mathematical three circle touching problem and morphological post-processing (*discs*, Figure 2). However, many apply simulation method from physics and engineering fields to create patterns resembling natural phenomena. For instance, realistic crack patterns were generated using a mesh simulation by iterative addition of new cracks (based on the highest priority material failure) and relaxation of the stress tensor in the mesh [136]. Lichen growth was simulated using a DLA-based model, including light and water flow simulation [137].

A very active related area of research in computer graphics is texture synthesis, i.e., production of textures similar to a given example (in some statistical sense). The generated image can then have useful properties the original lacks, such as being tileable (periodic). Impressive results have already been achieved [138,139,140]. If we have a sample of surface texture and wish to generate more samples, existing texture synthesis methods allow to do this with relative ease. The downside is, of course, the absence of physical interpretation of any texture properties. After all, these methods have been developed for visual impression, not physical accuracy. In general it is not possible to control physically interesting parameters of the surface (sticking coefficient, scaling exponent, etc.). However, there are cases when these techniques can be still useful even in a simulation, for example to render a much larger version of surface texture, which may be difficult to acquire otherwise.

A completely different approach to surface texture construction is the modification of existing data to enforce values of specific parameters. The basic case is the adjustment of value distribution to a prescribed one (*coerce*). It has been used for the creation of tunable random roughness for simulations [141], but also in the production of physical roughness standards [142]—in this case iteratively to ensure the produced standard conforms to the design. More complex iterative procedures can generate textures with multiple prescribed statistical parameters [143].

## 3. Synthetic Data Applications

### 3.1. Impact of Tip on SPM Results

**Figure 7:**
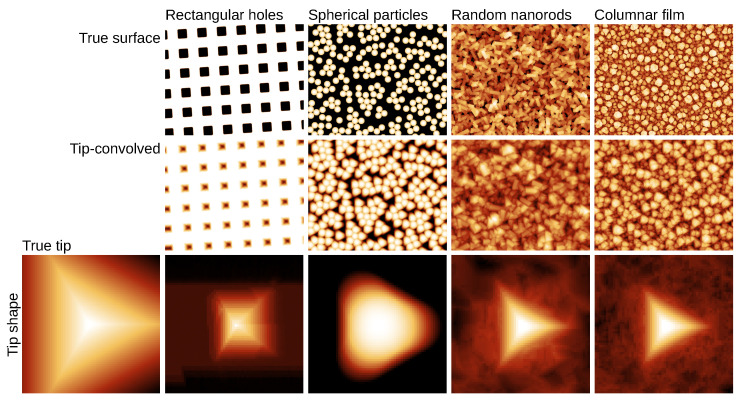
Tip convolution effect on different synthetic structures—2D grating, spherical nanoparticles, random nanorods and a columnar thin film. Top row: simulated data; middle row: data convolved with tip; bottom row: tip used for simulation and tips obtained using the blind estimation algorithm (enlarged; not in scale with the two upper rows).

**Figure 8:**
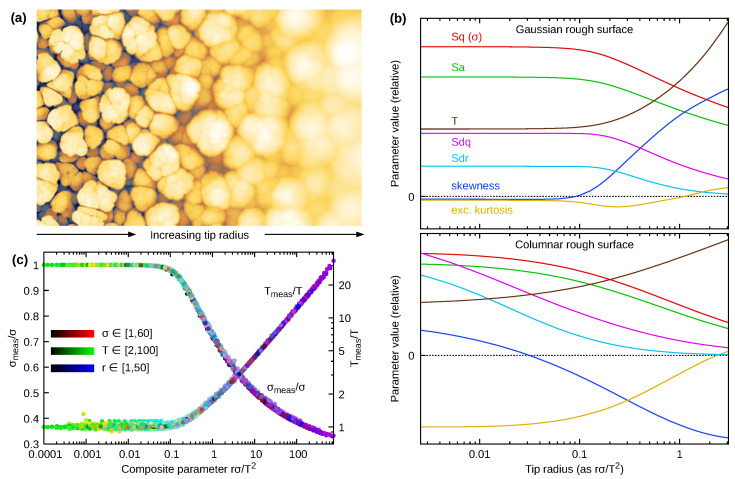
(**a**) Evolution of measured morphology of a columnar rough surface with increasing tip radius *r*. (**b**) Dependencies of selected roughness parameters surfaces on tip radius for Gaussian and columnar surfaces. (**c**) Ratios of measured values of σ and *T* to true values for Gaussian surfaces, plotted as functions of the dimensionless parameter $\sigma r/T^{2}$. Point RGB colours represent combinations of parameters σ, *T*, and *r*, as indicated.

Tip sharpness is a crucial factor of successful SPM measurements. Ideally, an infinitely sharp tip would provide undistorted image. Finite tip size leads to distortion and the resulting data are convolution (dilation) of the tip and surface shape [47]. Examples of these distortions can be seen in Figure 7 and Figure 8a (both simulated).

Since the tip can evolve during scanning due to wear and its radius determined previously or provided by the manufacturer is, therefore, no longer valid, it is necessary to estimate its geometry from data. Without synthetic data, it would be very hard to develop such estimation algorithms. By generating known data and known tip shape and by performing convolutions and tip estimations under different conditions we can analyse how the results are affected by influences like noise, feedback loop faults, or scan resolution.

As an example of such procedure, a radial basis neural network was trained using simulated data and then applied on scanned gratings data to reduce the influence of tip convolution [23]. It pointed out the problem of training the network, namely using suitable tip for training and also to points on surface where the information is lost. In Reference [22] neural networks were used to speed up the tip convolution impact analysis, namely to obtain a certainty metric to quantify the quality of local tip-sample contact. For this, rough surfaces with wide range of roughness parameters were generated. In Reference [24] the impact of tips having two asperities instead of one was investigated, focusing on analysis of fibril structures. The image blur related to tip convolution was estimated using Hough transform. Bayesian blind deconvolution was then used to remove it from the measured data. Synthetic data were used to demonstrate the versatility of the method for other types of surfaces, namely particles on a flat substrate.

An illustration of utilisation of synthetic data in tip convolution and blind estimation [47] is in Figure 7. Four synthetic topographies were generated, each with a different type of surface features. They were dilated by the same known tip and the results were used for blind tip estimation. The results are compared in the last row. The impact of surface character on the reconstructed shape is dramatic and shows how the lack of certain directions or slopes is reflected by the corresponding lack of tip geometry information in the convolved data.

Although the effect of tip convolution on direct dimensional measurements, like the width of a particle, can be almost intuitively understood, statistical quantities, like roughness parameters, are frequently impacted in a counter-intuitive way. Synthetic surfaces are invaluable for addressing this problem. Columnar thin films were synthesised in References [17,18] using ballistic deposition with limited particle relaxation. The goal of this procedure in Reference [17] was to better understand the growth-related roughening processes when conformal films are deposited on rough surfaces. Reference [18] studied the evolution of different statistical parameters of roughness when sample is convolved with tips of different radius, showing that the impact of wrong measurements on pores between individual columns using bigger tips has significant influence on both the height and lateral statistical quantities. A similar analysis was done for fractal surfaces [19], where limitations of fractal dimension analysis methods were identified, as well as large discrepancies between different analysis methods. Multi-fractal properties turned out to be even more complicated as tip convolution seems to add a sign of multi-fractal behaviour also to surfaces that were originally of mono-fractal nature [144]. Such analysis could not be done without using synthetic surfaces of known fractal properties. Synthetic particle deposition was used to estimate the reliability of different automated particle analysis methods in Reference [21], addressing the problems of analysis of nanoparticles on rough surfaces, where many of the segmentation methods can fail. It was found that the most critical are samples with medium particle coverage, where self-assembled arrays of particles have not yet developed (that could be analysed using spectral methods), but particles can no longer be treated as individual either.

Consider, now in more detail, the example of estimation of tip impact on statistical quantities of rough surfaces. The distortion of measured morphology depends on the tip radius *r* but also roughness character (in Figure 8a it is illustrated for synthetic columnar film data). The influence of roughness character was explored using simulated convolution with parabolic tips of varying radius for a standard Gaussian rough surface generated by spectral synthesis and a columnar rough film simulated using the *Columnar* deposition model. In both cases several 2400×2400 pixel images were generated, with correlation length T≈19px and rms roughness σ=1.85px. Tip convolution was simulated with tip apex radii covering more than two orders of magnitude, from very sharp to quite blunt. For all output images we evaluated the root mean square roughness (Sq), mean roughness (Sa), root mean square surface slope (Sdq), surfaces area ratio (Sdr), skewness, excess kurtosis, and correlation length (*T*), and averaged them over individual images.

The resulting dependencies are plotted in Figure 8b. Since the parameter ranges differ considerably and some are not even commensurable quantities, the curves were scaled to comparable ranges (preserving the relation between Sq and Sa). A few observations can be made. For the Gaussian surfaces, which are locally smooth, the parameters stay more or less at the true values up to a certain radius, and then they all start to deviate. In contrast, for the columnar film several quantities have noticeably non-zero derivatives even for the sharpest tip used in the simulation. This is the result of deep ravines in the topography, with bottoms inaccessible even by quite sharp tips. Some results are more puzzling, for instance the peculiar non-monotonic behaviour of kurtosis for Gaussian surface. We can also see that convolution with blunt tips makes the measured skewness of Gaussian surfaces positive. However, for columnar films, which are positively skewed, the measured skewness decreases and even becomes negative for blunt tips. Such effects would be difficult to notice without simulations.

This is even more true for the results of a larger-scale simulation with Gaussian rough surfaces, in which all *r*, σ and *T* were varied. Careful analysis revealed that the convolution problem is in fact characterised by just a single dimensionless parameter $\sigma r/T^{2}$. This is demonstrated in Figure 8c where the ratios of measured σ and *T* to true values are plotted as functions of $\sigma r/T^{2}$. It is evident that even though all three parameters varied over wide ranges the data form a single curve (for each σ and *T*). This result can be explained by dimensional analysis. When we scale the lateral coordinates by *b* and height by *c*, then *r*, σ and *T* scale by $c/b^{2}$, 1/b and 1/c, respectively. The only dimensionless number formed by *r*, σ and *T* which is preserved is $\sigma r/T^{2}$. However, the surface must scale like the tip to preserve their mutual geometrical relation. Meaning it needs to also be locally parabolic—which is satisfied by the locally smooth Gaussian surface (but not by other self-affine surfaces).

### 3.2. Levelling, Preprocessing, and Background Removal

Raw SPM data are rarely the final result of the measurements. In most cases they are evaluated to get a quantitative result, like size of nanoparticles, volume of grains, pitch of the grating or surface roughness. Preprocessing steps are usually needed for this, to correct the misalignment of the *z*-axis of the instrument to the sample normal, to correct the impact of drift or to remove scanner background. Synthetic data can be used to both develop the data processing methods and estimate their reliability or uncertainties.

**Figure 9:**
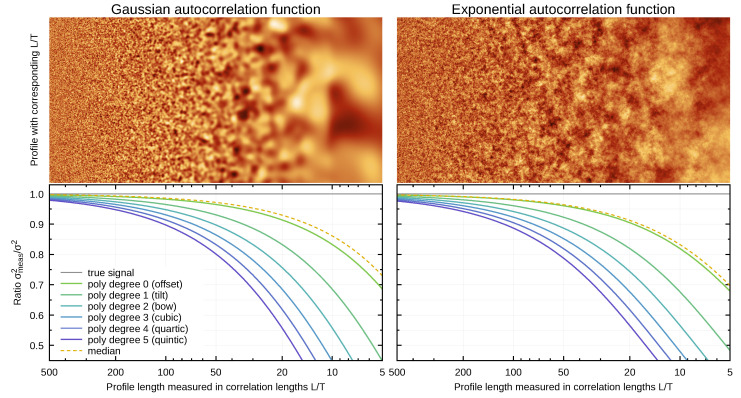
Ratio of measured roughness $\sigma_{\mathrm{meas}}^{2}$ to true value $\sigma^{2}$ depending on the ratio of profile length *L* and correlation length *T*. The bias is plotted for Gaussian and exponential autocorrelation and several common levelling types—subtraction of a few low-degree polynomials and the median. Vertical slices through the images illustrate the corresponding ratio L/T (image height is *L*).

An example of use of synthetic data for estimation of systematic errors in SPM data processing is the study of levelling-induced bias in roughness measurements [29,141]. Levelling is done as a pre-processing step in nearly all SPM measurements in many variations, from simple row mean value alignment up to polynomial background removal. Since the data are altered by levelling, it has impact on the roughness measurement results. Using synthetic data this impact was quantified, showing that in a large portion of SPM related papers the reported roughness values might be biased in range of tens of percents as the result of too small scan ranges. The problem is illustrated in Figure 9 for the mean square roughness σ and scan line levelling by polynomials. The ratio $\sigma_{\mathrm{meas}}^{2}/\sigma^{2}$ expresses how much the measured roughness is underestimated—ideally it should be 1. The underestimation is of course worse for shorter lengths *L*. However, it is clear that even for quite long scan lines (compared to the correlation length *T*), the roughness can be considerably underestimated, especially for higher polynomial degrees. A procedure for choosing a suitable scan range to prevent this problem already during the measurement is provided in Reference [141].

In Reference [26], methodology for using spectral density in the SPM data evaluation was studied. Synthetic data allowed discussion of various influences on its accuracy, including sampling, tip convolution, noise, and windowing in the Fourier transform. In Reference [27] the spectral density evaluation was extended to irregular regions, allowing the method to be used on grains or terraces covering only part of the SPM image. The method was validated using data generated by spectral synthesis.

Synthetic data were also used to test novel algorithms for using mono atomic silicon steps as a secondary realisation of the metre. Following the redefinition of the SI system of units the increased knowledge about silicon lattice spacing led to acceptance of this approach for SPM calibration [145]. To develop reliable methods for data levelling when tiny steps are evaluated from SPM data measured on large areas, synthetic data were used [30], namely to verify algorithms separating various scanning related errors like line roughness and scanner background from the sample geometry.

**Figure 10:**
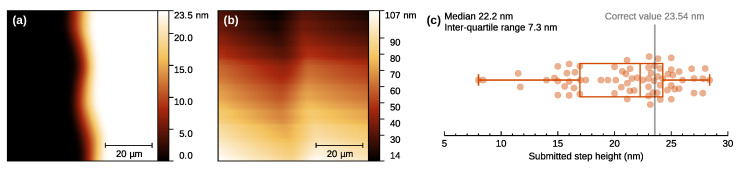
Study of user influence on SPM data evaluation: (**a**) The step to measure created using *pattern*—with smooth and somewhat wobbly edge, but otherwise ideal. (**b**) The image users actually received, with tilt and scanning artefacts added. (**c**) Distribution of user submitted evaluated step heights as a stripchart with jitter (the vertical offsets do not carry information; they only help to disperse the points) and a boxplot overlay.

In addition to the effect of concrete data pre-processing algorithms, there is also the freedom in which of them to use. Several paths can lead to similar results (e.g., images with aligned rows), but their impact on the data can be different. SPM users seldom choose based on rigorous criteria—the choice is more often the result of availability, discoverability, and habit. The impact of this user influence was studied in Reference [34] using combination of multiple data synthesis methods to create complex, but known data as illustrated on the step analysis example in Figure 10. A group of volunteers was then processing the data to determine the specified parameters, resulting in a rather worrying spread of determined values. Furthermore, data processing methods were classified on the basis of amount of user influence on them. It was done using an MC setup somewhat atypical for SPM as it included data processing steps carried out by human subjects. Batches of 100 synthetic images were generated, with known parameters—but unknown to the users. The generated images contained roughness, tilt, and defects, such as large particles. The users were then asked to level them as best they could, using levelling methods from prescribed sets. It was found that while humans are good at recognising defects (and marking them for exclusion from the processing), giving them more direct control over the levelling is questionable. The popular 3-point levelling method did not fare particularly well and humans also tended to over-correct random variations (although all levelling methods are guilty of this, even without user input [29,141,146]).

### 3.3. Non-Topographical SPM Quantities

So far the examples involved simulated topography. It is perhaps the most common case, but not a fundamental limitation. Any physical quantity measurable by SPM can be addressed given a physical model of the sample and a model of the probe-sample interaction, which can have different level of complexity.

As an example of use of simple tools applied to non-topographical data, magnetic domain data were simulated during development of methodology for tip transfer function reconstruction in magnetic force microscopy (MFM) [100] using the *phases* module. Since the purpose of the procedure was the estimation of an unknown function from non-ideal data, verification and quantification of systematic errors related to data processing using artificial data with added defects were key steps. Another major benefit of synthetic data was that virtual MFM data of any size and resolution could be used, even beyond what is feasible to measure.

Reference [100] used simulations to study and optimise several aspect of the reconstruction. The performance of different FFT window functions was compared using artificial data—with somewhat surprising results. Although FFT windows had been extensively studied for spectral analysis, their behaviour in transfer function estimation was not well known. The study found that beyond $C^{0}$ window smoothness and even shape did not play much role and the key parameter was the $L^{2}$ norm of window coefficients (and simple $C^{0}$ windows, such as Welch and Lanczos, are thus preferred). Simulations were also used to evaluate the influence of different regularisation parameter choices and to improve a procedure used to estimate the true magnetic domain structure from the measured image.

A very frequent use of synthetic data in non-topographical SPM measurements interpretation is related to understanding of topography artefacts in the other quantities channels. Artefacts related to sample topography can be found in all the regimes (electric, magnetic, thermal, mechanical, and optical) and belong among the largest uncertainty sources when other quantities are evaluated.

Synthetic topographical data are only the first step. A physical model of the interaction has to be formulated and we must simulate the signal generation process. This can be for example finite element method (FEM) modelling which was used for simple topographic structures to simulate their impact on Kelvin Force Probe microscopy in Reference [147]. Here it was found that the topography impact on surface potential measurements is relatively small. This, however, is not the case in many other SPM techniques and topography artefacts can dominate the signal. Simple 1D synthetic structures were used to simulate topography artefacts in aperture-based scanning near field optical microscopy [148], where a model based on calculating the real distance of the fibre aperture from surface was used. Even if the probe follows trajectory preserving a constant distance to the surface, this keeps constant the shortest distance from any point on the probe to the surface. However, the distance between surface and the aperture that is in the centre of the probe apex varies when scanning across topographic features (e.g., when following a step). Combined with the fast decay of the evanescent field, it produces topography artefacts in the optical signal. More complex analysis was done using 2D synthetic patterns in Reference [149], where Green’s tensor technique was used to calculate the field distribution in the probe-sample region, showing that it is easy to misinterpret topographical contrast as dielectric contrast (which is the target measurand). Topography artefacts were also modelled in the scattering type scanning near field optical microscopy, which is an even higher resolution optical SPM technique [150]. For this, a simple synthesised topographic structure representing a nanopillar array was combined with an idealised scattering tip and the interaction was handled using a dipole–dipole theoretical model. This allowed examination of different aspects of far-field suppression using higher harmonic signals.

**Figure 11:**
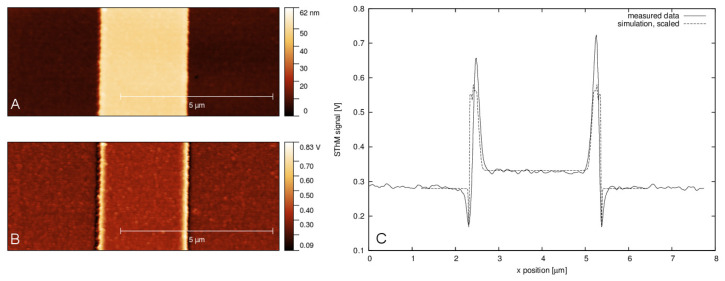
Topography artefacts in SThM: (**A**,**B**) topography and thermal signal on a step height structure, (**C**) experimental data and result of the FDM calculation using simulated step height structure [36], simulating a single profile. Simulated signal is scaled to match the raw SThM signal coming from the probe and the Wheatstone bridge.

Additionally, in scanning thermal microscopy (SThM), the topography artefacts can dominate the signal and methods for their simulation are needed [36]. In Figure 11, the typical behaviour of the thermal signal on a step edge is shown, together with the result of a finite difference model (FDM) solving the Poisson equation. Synthetic data were used here to create the simulation geometry, taking the simulated surface and probe from Gwyddion, converting it to a rectangular mesh, and calculating the heat flow between probe and sample. The process was repeated for every tip position, producing a virtual profile (or virtual image in the 2D case). Synthetic data were used to create simple structures that could be compared to experimental data, as shown in the figure, and were an important step in validation of the method and moving towards simulations of more realistic structures.

Finally, numerical analysis can be used for the non-topographical data interpretation, modelling the probe-sample interaction using a structural model of the sample and creating virtual images that are compared to real measurements. During development and testing of the numerical models used for these purposes various synthetic datasets are also frequently used. As an example, spectral analysis of measured surface irregularities was studied in Reference [151], where STM signal was simulated for synthetic nanoparticle topographies. The results were used to assist in the interpretation of data coming from different laboratories and affected by different imperfections, like noise and feedback loop effects.

More complex examples include models for addressing mechanical response in nano-mechanical SPM regimes were developed using synthetic data. Such methods have potential of sub-surface imaging on soft samples, which however needs advanced data interpretation methods. Numerical calculations based on a synthetic structural model and FEM was used to interpret data measured on living cells [152] when responding to external mechanical stimuli.

In general, physical models of probe-sample interaction can be quite complex and simulation of SPM data can be a scientific area on its own. For example, quantum-mechanical phenomena need to be taken into account when very high resolution ultra-high vacuum measurements are interpreted, which has been done using a virtual non-contact AFM [153]. Simulations at this level are however beyond the scope of this paper which focuses on synthetic data that can be easily generated, e.g., using Gwyddion open source software and then used for various routine tasks related to SPM data processing.

### 3.4. Use of Synthetic Data for Better Sampling

Synthetic data can also be used for development of better sampling techniques or techniques that allow fusion of datasets sampled differently. Traditionally SPM data are sampled regularly, forming a rectangle filled by equally spaced data points lying on a grid. However, the relevant information is usually not distributed homogeneously on the sample. Some areas are more important than others for the quantities that we would like to obtain from the measurement. Development of better sampling can focus on better treatment of steep edges on the surface [154], reduction in the number of data points via compressed sensing [155], or better statistical coverage of roughness [8].

As an example of synthetic data use in this area, in Reference [33] randomly rough surfaces with exponential autocorrelation function were generated to simulate measurements with low and high density sampling. Gaussian process based data fusion was then used and tested on these data. Using this method, the simulated datasets could be merged, when the accuracy of the low and high density measurements was different, which is often encountered in practice. Data fusion results were then compared with synthetic data that were used to create the input sets, making the analysis of method performance straightforward.

**Figure 12:**
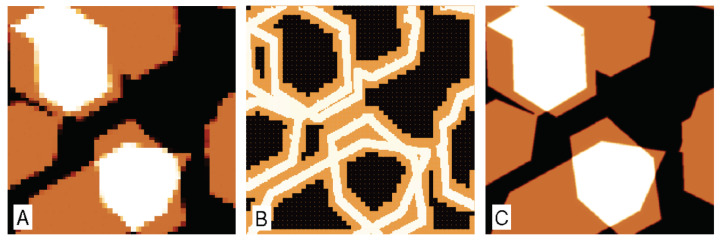
Scan path refinement while simulating measurement on 2D material flakes: (**A**) coarse topographical measurement; (**B**) refinement paths—colour indicates measurement point density (in the dark regions individual points can be seen as dots); (**C**) XYZ data from all three measurements merged together.

Synthetic data were also used for the development of methods for generation of non-raster scan paths in the open source library Gwyscan [8], focusing on scan paths that would better represent the statistical nature of samples, e.g., by addressing larger span of spatial frequencies while measuring the same number of points as in regularly spaced scan. Use of general XYZ data instead of regularly spaced samples allows creation of more advanced scan paths, measuring only relevant parts of the sample. An example of scan path refinement is shown in Figure 12. Here, data similar to flakes of a 2D material were generated and the simulated scans were covering an area of 5×5 μm. The coarsest image (see Figure 12A) with only 50×50 points laterally spaced by 100 nm was used to create the next refined path, based on the local sample topography variance. The refined path points were laterally spaced by 10 nm and were used to create a next refinement, laterally spaced by 1 nm. At the end, all the XYZ points were merged and image corresponding to the desired pixel size of 1 nm was obtained, in total measuring only about 25% of points compared to a full scan.

## 4. Discussion

In the previous section we recapitulated various styles and strategies for use of synthetic data. It can be seen that the methodology varies from task to task. The unifying idea is to simulate how an effect related to SPM measurement or data processing alters the data, which can be done best using known data. Generation of the data involves randomness in most cases and resembles the use of Monte Carlo (MC) in uncertainty estimation. The MC approach is used in metrology when measurement results cannot be formulated analytically, using an equation (which would be differentiated for the error propagation rule to get the uncertainty contributions of different input quantities). MC is an alternative in which input quantities are generated with appropriate probability distributions and the result is computed for many of their combinations, forming the probability distribution of the results. More guidance is provided in the guide to the expression of uncertainty in measurement (GUM) [156]. The method then provides both the result and its uncertainty.

Sometimes the procedure applied to synthetic data can differ quite a lot from how we imagine the typical MC, but still be built using the same principles. For instance, in a part of the user influence study [34], large amounts of data were generated and then processed by experienced Gwyddion users manually. The goal was to see how the user’s choice of algorithms and their parameters influences the results (see also Section 3.2). Therefore, a human was the ‘instrument’ here. However, for the rest the MC approach was followed.

It should be noted that even in a classical uncertainty estimation MC may not be the most efficient computational procedure. If there are only a handful of uncertain or variable input parameters, non-sampling methods based on polynomial chaos expansion [157] can be vastly more efficient. Their basic idea is the expansion of output parameters as functions of input parameters in a polynomial basis, with unknown coefficients. Determining the coefficients then establishes a relation between input and output parameters and their distributions [158]. In so-called arbitrary polynomial chaos the technique is formulated only in terms of moments of the distributions, avoiding the necessity of postulating specific distributions and allowing data-driven calculations [159]. However, huge numbers of random input parameters—quite common in the SPM simulations—present a challenge for polynomial chaos and using MC can have significant benefits.

More importantly, standard uncertainty analysis may be exactly what we are doing and GUM exactly the appropriate methodology to follow, though it also may not be. We need a more nuanced view on procedures described collectively as ‘generate pseudorandom inputs and obtain distributions of outputs’. Consider what would be the result of running the simulation with infinitely large data. There are two basic outcomes: an infinitely precise value and nothing. An example of the former is the study of tip convolution in Section 3.1. In the limit of infinite image, tip convolution changes the surface area of a columnar film (for instance) by a precise amount, independent on the sequence of random numbers used in the simulation, under an ergodicity assumption. In fact, the images used in Section 3.1 were already quote close to infinite from a practical standpoint. The relative standard deviations of the parameters in Figure 8b were around $10^{-3}$ or smaller, i.e., a single MC run would suffice to plot the curves.

This gives context to the large number of MC runs suggested by GUM (e.g., $10^{6}$). For the surface part, more sampling of the probability space can be done either by generating more surfaces—or by generating larger ones. The second is usually more efficient and also reduces the influence of boundary regions that can cause artefacts. Large images do not help if tip radius is uncertain (although here polynomial chaos could be utilised). Therefore, we should use the adaptive approach, also suggested in GUM, increasing surface size up to the moment when statistical parameters of the result converge.

How is the hypothetical sharp value we would obtain from an infinite-image simulation related to uncertainties? It may be the uncertainty (more precisely, its systematic part) if we simulate an unwanted effect which may be left uncorrected and we attempt to estimate the corresponding bias. The hypothetical sharp value may also be simply our result—and then we would like to know its uncertainty. This brings us to the second possible outcome of infinite-image simulation, nothing. An example, polynomial background subtraction, was discussed in Section 3.2. Subtraction of polynomials has no effect in the limit of infinitely large flat rough surfaces for any fixed polynomial degree. Similarly, the MFM transfer function could be reconstructed exactly given infinite data, more or less no matter how we do it (Section 3.3). In this case we study the data processing method itself and its behaviour for finite measurements. It is essential to use image sizes, noise, and other parameters corresponding to real experiments. The distribution of results is tied to image size. Scaling results to different conditions may be possible, but is not generally reliable especially if boundary effects are significant. Both biases and variances frequently scale with $T^{2}/A$, where *A* is image area and *T* correlation length or a similar characteristic length—not as 1/N with the number of image pixels *N*— and size effects can be considerable even for relatively large images [29,141,146].

Several practical points deserve attention when using artificial data. Running simulation and data processing procedures manually and interactively is instructive and can lead to eye-opening observations. Proper large-scale MC usually still follows, involving the generation of many different surfaces, SPM tips, and other objects. This would be very tedious if done manually. Most Gwyddion functionality is being developed as a set of software libraries [6], with C and Python interfaces. This allows scripting the procedures, or even writing highly efficient C programs utilising Gwyddion functions. This is also how most of the examples were obtained.

The same input parameters must produce identical artificial surfaces across runs—but also operating systems and software versions. For geometrical parameters this is a requirement for using the models with non-intrusive polynomial chaos methods. Most models have random aspects, ranging from simple deformations and variations among individual features to stochastic simulations consuming a stream of random numbers. The sequence of numbers produced by a concrete pseudorandom generator is deterministic and given by the seed (initial state).

However, reproducing a number sequence is not always sufficient. A stronger requirement is that a small change of model parameter results in a small change of the output, at least where feasible (it is not possible for simulations in the chaotic regime, for instance). In other words, the random synthetic data evolve continuously if we change parameters continuously. This is achieved by a combination several techniques, in most cases by (a) using multiple independent random sequences for independent random inputs; (b) if necessary, throwing away unused random numbers that would be used in other circumstances; and (c) filling random parameters using a stable scheme, for instance from image centre outwards. Examples of continuous change of output with parameters can be seen in Figure 9. Each column of the simulated roughness image came from a different image (all generated by spectral synthesis). They could be joined to one continuous image thanks to the generator stability.

Theoretical modelling of SPM data is an active field. Much more is going on which lies out of the focus of this work. For example detailed atomistic models are now common in STM [52,53,54] or interaction with biomolecules and biological samples [160]. Every sample and each SPM technique has its own quirks and specific modelling approaches [3]. The methods discussed here do not substitute the models that are related to physical mechanisms of the data acquisition in SPM. However, they can be used to feed them with suitable datasets like in our SThM work [36] where Gwyddion data were directly used to create the mesh for FDM calculations.

## 5. Conclusions

Use of synthetic data can not only significantly save time when evaluating uncertainties in quantitative SPM, but can also allow analysis of individual uncertainty components that would be otherwise jumbled together if only experimental data were used. This helps with improvement of the quantitative SPM technique from all points of view: data collection (e.g., compressed sensing), processing of measured data (e.g., impact of levelling and other preprocessing) and even basic understanding of phenomena related to the method (e.g., tip convolution). In all these areas the generation of reliable and well understood synthetic datasets representing wide range of potential surface geometries is useful, as demonstrated in this paper. The data reliability here means that the synthetic data should be deterministic and predictable (at least in the statistical sense) and should be open for chaining them to simulate multiple effects. The demonstrated implementations of discussed algorithms in Gwyddion have these properties.

Another benefit of using synthetic data is its suitability for testing and comparing different algorithms performance during the data processing software development. This is often done on basis of real SPM data in the literature as authors want to demonstrate practical applicability of the algorithms on realistic data. However, as is discussed in this paper, the data synthesis methods are already so mature that known data that are very similar to real measurements can be generated and different SPM error sources can be added to them in a deterministic way. This can make the software validation much easier than in the case of experimental data with all influences fused together. Even further, whole software packages can be compared and validated on basis of synthetic data.

## Data Availability

The software used to generate all the data [7] is publicly available under GNU General Public License, version 2 or later.

## Abbreviations

The following abbreviations are used in this manuscript:

AFM	Atomic Force Microscopy
C-AFM	Conductive Atomic Force Microscopy
DDA	Deposition, Diffusion and Aggregation
DFT	Density Functional Theory
DLA	Diffusion-Limited Aggregation
EDT	Euclidean Distance Transform
FDM	Finite Difference Model
FEM	Finite Element Method
FFT	Fast Fourier Transform
GUM	Guide to the expression of Uncertainty in Measurement
KLT	Kessler–Levine–Tu
KPZ	Kardar–Parisi–Zhang
LALI	Local Activation and Long-range Inhibition
MC	Monte Carlo
MEMS	Microelectromechanical systems
MFM	Magnetic Force Microscopy
PDE	Partial Differential Equation
PID	Proportional-Integral-Derivative
RGB	Red, Green and Blue
SPM	Scanning Probe Microscopy
SThM	Scanning Thermal Microscopy
STM	Scanning Tunnelling Microscopy
